# The Impact of Secure Attachment on Internet Altruistic Behavior: From the Helper and Seeker’s Perspective

**DOI:** 10.3390/bs16060877

**Published:** 2026-06-01

**Authors:** Lijuan Huang, Xianliang Zheng, Qingfeng Qiu

**Affiliations:** 1 Mental Health Center, Jiangxi Institute of Technology, Nanchang 330022, China; huohuohaha@jxit.edu.cn; 2 School of Educational Science, Gannan Normal University, Ganzhou 341003, China; 3 Personnel Office, Jiangxi Institute of Technology, Nanchang 330022, China; heui2@jxut.edu.cn

**Keywords:** internet altruistic behaviors, secure attachment, secure attachment priming, positive empathy, emoji symbols

## Abstract

Previous research on Internet altruistic behavior (IAB) has primarily focused on helpers. However, as a form of prosocial behavior embedded in online interpersonal interaction, IAB is likely shaped by characteristics of both helpers and seekers. Accordingly, the present research integrated attachment theory and social information processing models to examine how helper-related factors (secure attachment and positive empathy) and seeker-related factors (emoji symbols) influence IAB, as well as the mechanisms underlying these effects. Three studies were conducted. Study 1 used a one-factor between-subjects design to examine the effect of secure attachment on IAB. Study 2 employed an experimental causal-chain approach with three sub-studies to test the causal links among secure attachment, positive empathy, and IAB. Study 3 used a 2 (positive empathy: high vs. low) × 2 (emoji: present vs. absent) between-subjects design to examine whether seekers’ emoji use moderated the relationship between helpers’ positive empathy and IAB. The results showed that individuals with higher (vs. lower) secure attachment engaged in more IAB, and that positive empathy mediated the relationship between secure attachment and IAB. In addition, seekers’ emoji use significantly moderated the relationship between helpers’ positive empathy and IAB. Specifically, this moderation was antagonistic: the presence of emoji was associated with a weaker, rather than stronger, positive relationship between positive empathy and IAB. Nevertheless, overall levels of IAB were higher in messages containing emoji. These findings provide a more comprehensive understanding of the antecedents of IAB and offer practical implications for promoting altruistic behavior in online contexts.

## 1. Introduction

In the 21st century, the Internet has become a central context for social interaction. In China in particular, online environments increasingly shape how individuals communicate, seek support, and build relationships. Internet altruistic behavior (IAB), defined as voluntarily providing unreciprocated help online (e.g., offering advice or information; Zheng et al., 2021), reflects this shift and has attracted scholarly attention because of its role in fostering supportive interpersonal connections (Nicolas, 2008).

Attachment theory suggests that early relational experiences influence later prosocial tendencies, and secure attachment has been linked to greater altruism in offline contexts (Pan et al., 2019). However, relatively little research has examined whether secure attachment also promotes IAB, a form of prosocial behavior enacted in digital settings. This question is particularly relevant among university students, who are deeply embedded in diverse online environments and whose behavioral patterns often reflect emerging digital trends (Salwan et al., 2026; Trant et al., 2026). Understanding how secure attachment shapes online helping behaviors among young adults may therefore have practical value for promoting healthy and prosocial engagement in complex online contexts.

Existing research has primarily focused on helper-related traits (e.g., self-esteem, shyness) as predictors of IAB (Erreygers et al., 2018). Yet IAB arises through interactions between helpers and seekers, suggesting that seeker-related cues may also play an important role in shaping helping decisions. In particular, online communication frequently relies on emoji to convey emotions and intentions, but their potential influence on altruistic responding remains largely unexplored.

Accordingly, the present study adopts a dual-perspective framework. First, we examine how helper-related factors (secure attachment and positive empathy) facilitate IAB. Second, we investigate whether seekers’ use of emoji moderates this process. By simulating realistic online help-seeking scenarios, this research aims to clarify how attachment security, empathy, and emoji-based social cues jointly shape IAB, thereby extending both helper-centered and seeker-centered perspectives on online prosocial behavior.

### 1.1. Secure Attachment and IAB

Helper-related factors play an important role in predicting IAB. Prior research has shown that characteristics such as self-esteem are positively associated with IAB (Zheng et al., 2021). However, comparatively less attention has been paid to secure attachment, despite evidence that it promotes prosocial behavior more broadly (Gross et al., 2017). Attachment refers to individuals’ tendency to seek closeness, comfort, and protection from significant others (e.g., parents or romantic partners; Bowlby, 1969). Secure attachment develops through consistent and supportive early caregiving experiences, which foster positive internal working models of the self and others (Bowlby, 1969). As a result, securely attached individuals tend to display more adaptive cognitive styles, healthier emotional functioning, and more constructive social behaviors than insecurely attached individuals (Newman-Taylor et al., 2021).

In addition, SAP may enhance the activation and accessibility of secure attachment representations within long-term memory networks, thereby promoting cognition, emotion, and behavior that are consistent with attachment security (Jones et al., 2022). Prior studies have shown that SAP increases feelings of security (Hudson & Fraley, 2018) and facilitates positive interpersonal behaviors, including prosocial responding and self-disclosure (de Bruijn et al., 2022). Accordingly, SAP provides a useful experimental tool for examining the causal effects of temporary attachment security.

According to attachment theory, individuals’ relationships with attachment figures are internalized into working models that guide interpersonal behavior (Bowlby, 1973). These models include representations of both the self and others. Under conditions of greater attachment security, individuals are more likely to perceive others as trustworthy and worthy of support, while also viewing themselves as capable of helping effectively (Shaver et al., 2016). Consistent with this view, SAP has been linked to more trust and less hostility in interpersonal relationships (Mikulincer & Shaver, 2001), as well as more positive responses toward others and social situations (Baldwin et al., 1996). In online contexts, such tendencies may increase willingness to attend to others’ needs and provide assistance. In addition, secure attachment may strengthen prosocial values such as kindness and universalism (Pan et al., 2019), thereby further promoting altruistic behavior in cyberspace. Taken together, these perspectives suggest that secure attachment should facilitate IAB. Therefore, we hypothesized that individuals exposed to SAP would exhibit more IAB than those in the control condition.

### 1.2. Mediating Role of Positive Empathy

From the helper’s perspective, secure attachment may represent a proximal predictor of IAB. However, the mechanisms through which secure attachment promotes IAB remain unclear. In recent years, with the rise of positive psychology, positive empathy has received increasing scholarly attention. Positive empathy refers to the ability to perceive, share, and respond to others’ positive emotional experiences (Yue et al., 2021a). Empathizing with others’ positive emotions is an important aspect of social life and plays a valuable role in maintaining harmonious relationships and interpersonal functioning (Liu et al., 2022; Yue et al., 2021b). Thus, like general empathy, positive empathy serves an important social function (Yue et al., 2021a). According to the mood maintenance hypothesis (Mischel et al., 1973), individuals experiencing positive affect are motivated to engage in positive behaviors in order to sustain that state. In this context, the positive emotions experienced by helpers (e.g., positive empathy) may serve as an intrinsic motivation for prosocial actions such as IAB (Aknin et al., 2012). Moreover, engaging in IAB may itself help individuals maintain positive psychological states, such as pleasure and satisfaction (Zheng & Zhao, 2015). Because IAB is fundamentally similar to offline prosocial behavior (Zheng et al., 2021), we expected that helpers with higher (vs. lower) levels of positive empathy would exhibit more IAB.

Furthermore, positive empathy may arise as an emotional consequence of secure attachment. On the one hand, SAP has been shown to effectively elicit positive emotions (Clear & Zimmer-Gembeck, 2017). Neurocognitive studies have further demonstrated that SAP activates multiple brain regions associated with positive emotional experiences (Canterberry & Gillath, 2013). On the other hand, individuals exposed to SAP tend to show greater empathic concern (Cassidy et al., 2018). Accordingly, SAP may simultaneously enhance empathy and promote more positive emotional experiences (Bryant & Bali, 2018), thereby increasing positive empathy.

The broaden-and-build cycle of attachment security (Mikulincer & Shaver, 2007) also provides a useful framework for understanding the mediating role of positive empathy. According to this perspective, SAP produces beneficial changes in affect, cognition, and behavior. First, SAP may reduce negative emotions while increasing positive emotions (e.g., positive empathy), thereby generating feelings of warmth and pleasure. Second, secure attachment representations and positive affective experiences may broaden individuals’ views of themselves and others, such as believing that they are capable of helping and that others are trustworthy. Finally, guided by these positive emotional and cognitive states, helpers may be more likely to engage in prosocial actions such as IAB. Therefore, positive empathy may mediate the association between secure attachment and IAB.

### 1.3. Moderating Role of Emoji

As a growing feature of modern online interaction, emojis have become increasingly prevalent in social networking sites and digital communication (Novak et al., 2015). Derived from the Japanese words “e” (picture) and “moji” (character), emojis include facial expressions, gestures, objects, animals, and other visual symbols that can convey semantic and emotional information beyond plain text (Scheffler et al., 2022). In non-face-to-face online environments, emojis have become an important component of interpersonal communication, enriching digital interactions and creating more expressive forms of social exchange. Previous studies have shown that emojis can facilitate online interaction, increase perceived warmth, and strengthen emotional connections between message senders and receivers (Boutet et al., 2021; Pfeifer et al., 2022). Compared with plain text, messages containing emojis are often perceived as more pleasant and socially appealing (Boutet et al., 2021; Riordan, 2017). Neurophysiological evidence further suggests that emojis can elicit pleasurable emotional experiences (Gantiva et al., 2021) and empathic responses (Liao et al., 2021), thereby influencing individuals’ online behavioral responses (Zhang et al., 2021). Accordingly, whether seekers use emojis in help-seeking messages may influence helpers’ altruistic behavior.

Emojis are frequently used to communicate emotional states such as happiness, amusement, or anger (Chen et al., 2017). Thus, the use of emojis may help individuals express emotions more effectively and enable others to recognize these emotions more easily. In everyday interactions, individuals seeking help are often more likely to express politeness, gratitude, and positive emotions. Prior research has shown that when helpers perceive such positive emotional expressions from seekers, their prosocial motivation may increase (Rand et al., 2015). Moreover, the wide variety and visual expressiveness of emojis may compensate for the limitations of text-based communication in conveying appreciation and warmth in cyberspace.

The social information-processing model (Crick & Dodge, 1994) further suggests that prosocial behavior develops through several stages, including cue encoding, interpretation, response generation, and behavioral enactment. When helpers detect social cues from seekers (e.g., emojis), they may interpret these cues and subsequently adjust their behavioral responses (e.g., IAB). Specifically, individuals high in positive empathy may be especially sensitive to help-seeking messages containing emojis. The visual and emotional characteristics of emojis may make seekers’ emotions (e.g., gratitude) easier to perceive, while also increasing the enjoyment of communication (McShane et al., 2021) and reducing psychological distance between helpers and seekers (Zhang et al., 2021). These processes may, in turn, facilitate helping behavior. In contrast, plain-text help-seeking messages without emojis may provide fewer emotional cues, making them less engaging and potentially less likely to elicit emotional resonance or empathic responses (Pfeifer et al., 2022; Riordan, 2017; J. Y. Yang et al., 2021). Therefore, we proposed that emojis would moderate the relationship between positive empathy and IAB, such that the positive association between positive empathy and IAB would be stronger when emojis were present.

### 1.4. The Present Study

IAB has attracted increasing scholarly attention. However, several important gaps remain in the existing literature. First, prior studies have relied predominantly on correlational designs, leaving the causal mechanisms underlying IAB insufficiently examined. Second, previous research has focused primarily on helper-related factors while paying comparatively less attention to the role of seekers within online helping interactions. Third, although IAB occurs in digital environments, relatively little research has explored how online contextual cues—such as emojis commonly used in computer-mediated communication—may shape altruistic behavior. To address these gaps, the present research examined whether activating helpers’ sense of secure attachment would promote IAB (Study 1), whether positive empathy would mediate the relationship between secure attachment and IAB (Study 2), and whether seekers’ use of emojis would moderate the relationship between positive empathy and IAB (Study 3). Overall, this study aimed to investigate the influence of secure attachment on IAB and its underlying mechanisms from the dual perspectives of helpers and seekers (see Supplementary Materials Figure S1). The hypotheses were as follows:

**Hypothesis** **1.** *Individuals exposed to SAP will exhibit more IAB than those in the control condition.*

**Hypothesis** **2.** *Positive empathy will mediate the relationship between secure attachment and IAB.*

**Hypothesis** **3.** *Emojis will moderate the relationship between positive empathy and IAB, such that the positive association between positive empathy and IAB will be stronger when emojis are present.*

## 2. Study 1: Do People with High-Security Attachments Engage in More IAB?—From a Helper’s Perspective

Study 1 aimed to examine whether individuals with experimentally activated secure attachment would exhibit greater IAB. We hypothesized that participants in the SAP condition would exhibit higher levels of IAB than those in the non-SAP condition (H1). Study 1 utilized a one-way between-subjects design in which secure attachment (SAP group/non-SAP group) was the independent variable and the dependent variable was IAB.

### 2.1. Participants

An a priori power analysis conducted using G*Power 3.1 indicated that a minimum sample size of 128 participants would be required to detect a medium effect size (d = 0.50) with 80% power at α = 0.05 (two-tailed). Participants were undergraduate students recruited through convenience sampling from public courses at a comprehensive university in Jiangxi Province, China. Inclusion criteria were as follows: (a) age 18 years or older, (b) self-reported normal or corrected-to-normal vision, and (c) at least 1 h of daily Internet use to ensure sufficient online interaction experience. Participants were excluded if they failed to provide complete demographic information, left a substantial number of items unanswered, or showed obvious response patterns.

A total of 200 paper questionnaires were distributed. After excluding nine invalid responses, the final sample consisted of 191 participants (101 men, 52.88%). Participants were randomly assigned to either the SAP group (N = 97) or the non-SAP group (N = 94). The mean age was 19.20 years (SD = 0.91), the mean Internet-use history was 7.64 years (SD = 2.63), and the average daily Internet use was 8.35 h (SD = 3.20). All participants provided written informed consent and received compensation upon completion of the study. The study protocol was approved by the Ethics in Human Research Committee of the authors’ university.

### 2.2. Measures

#### 2.2.1. SAP

Referring to a related study (Mikulincer & Shaver, 2001), we used the recall writing task paradigm to initiate participants’ secure attachment. Participants were randomly assigned to either the SAP or non-SAP condition. The material for the priming group was: Please recall a person close to you who is very intimate with you, it can be a family member, a lover, or a dear friend, and you feel comfortable and warm with him/her. When you encounter something happy, you first want to share it with him/her. Meanwhile, when you encounter difficulties and frustrations, you want to be the first to talk to his/her and get his/her help.

The material for the non-SAP group was: Please recall a person around you with whom you have an ordinary relationship, he/she may be one of your many relatives, or one of your classmates, but you are not very close to him/her, but you don’t dislike him/her either. When you encounter joyful things, you will not remember to share with him/her, when you meet difficulties and frustrations, you will not take the initiative to seek his/her help.

After reading the materials in either the SAP or non-SAP group, participants were asked to answer 5 questions (e.g., “What do you imagine the person’s relationship with you to be like?”). (Note: These five questions were only used to help participants recall and were not included as study variables in the post-analysis.) After completing the writing task, participants were instructed to imagine that the recalled person was physically present beside them.

Referring to previous studies (Mikulincer & Shaver, 2001), we collected five words that express feelings of secure attachment: warm, caring, supportive, secure, and intimate. Participants were asked to rate five questions based on how they felt about the attachment figure when they were with them after recalling the writing task, using a 5-point scale (1 = very little, 5 = very much). For instance, “The level of warmth I feel when I imagine him/her beside me”. The Cronbach’s α in this study was 0.96.

#### 2.2.2. Writing Validity

According to the experimental manipulation of existing research (Mikulincer & Shaver, 2001), after participants completed a recall writing task, writing vividness and ease (i.e., vividness/ease of writing during the recall process) were measured. Items were scored on a 5-point scale (1 = less vividness/easy, 5 = very vividness/easy).

#### 2.2.3. IAB

The IAB scale developed by Zheng et al. (2011) was adapted. The scale contains 26 items, including four dimensions: Internet guidance (e.g., giving answers and guidance to questions asked online), Internet support (e.g., giving concern and encouragement to Internet users), Internet reminding (e.g., consciously posting to remind people after being scammed online), and Internet sharing (e.g., sharing one’s own successful learning experience with others online). The scale was scored on a 4-point scale, with higher scores indicating higher levels of IAB. The Cronbach’s α in this study was 0.93.

### 2.3. Procedures

After entering a quiet, comfortable classroom, participants were randomly assigned to two groups (SAP/non-SAP) and first completed a demographic variable survey followed by a recall writing task. After this, participants were instructed to imagine that the recalled person was physically present beside them. Next, participants took the SAP validity measure, completed the writing vividness and ease measures, and finally completed the IAB scale. At the end of the experiment, participants received compensation for their participation. All study materials and datasets for Studies 1–3 are publicly available on the Open Science Framework (OSF): https://osf.io/ynh5k/?view_only=6a35f705a26046a7a5691dcdfe05aea1 (accessed on 20 May 2026).

Informed Consent Statement. Informed consent was obtained from all participants. Written consent was acquired via a consent form signed before study participation. The consent process was conducted by the research team. Consent was obtained between 1 March 2023, and 31 March 2024, from adult participants (mainly college students), who were informed that their involvement was voluntary, that they could withdraw at any time without penalty, and that their anonymized responses would be used for research purposes only. The study involved no vulnerable populations. All identifiable personal information was removed, and data were stored securely and accessed only by the research team.

### 2.4. Results

#### 2.4.1. Preliminary Analyses

Independent sample *t*-test revealed a non-significant difference between the SAP group and the non-SAP group for ease of writing (*t*(189) = 0.94, *p* = 0.35) and vividness of writing (*t*(189) = 1.65, *p* = 0.10). This indicated that there was no significant difference in the effectiveness of the writing task between the two groups. In addition, the results showed that the manipulation of the helper’s secure attachment was valid: there was a significant difference between the SAP group (*M* = 4.09, *SD* = 0.81) and the non-SAP group (*M* = 2.26, *SD* = 0.57), *t*(189) = 18.22, *p* < 0.001, Cohen’s d = 2.63. This large effect size suggests that the autobiographical recall manipulation created a strong contrast between the two attachment conditions, although such effects should be interpreted cautiously given the possibility of heightened demand characteristics in experimental priming paradigms.

Furthermore, to ensure adequate statistical power, an a priori sensitivity power analysis was conducted using GPower 3.1. Setting α = 0.05 and power (1 − *β*) = 0.80, and with the obtained sample sizes (N-SAP = 97, Nnon-SAP = 94), the analysis indicated that the independent-samples *t*-test design was sufficiently sensitive to detect a minimum effect size of Cohen’s d = 0.41.

#### 2.4.2. Hypothesis Testing

**Figure 1:**
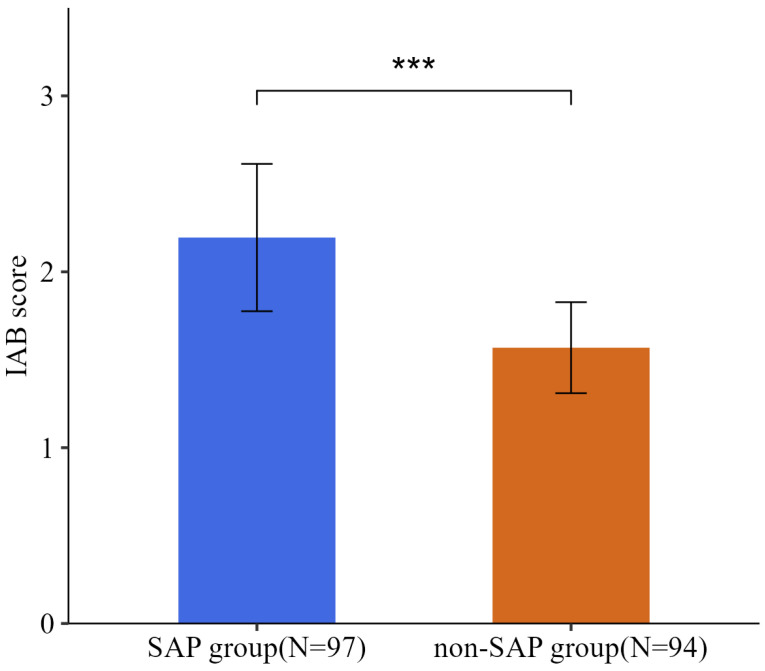
Differences in IAB between SAP group and non-SAP group. Note. *** *p* < 0.001.

The results of independent sample *t*-test showed that the IAB of SAP group (*M* = 2.21, *SD* = 0.44) was significantly higher than that of non-SAP group (*M* = 1.57, *SD* = 0.26), *t*(189) = 12.28, *p* < 0.001, Cohen’s d = 1.77, supporting Hypothesis 1. The results are shown in Figure 1.

## 3. Study 2: How Does Secure Attachment Affect IAB?—From a Helper’s Perspective

Study 2 further examined the mechanisms through which secure attachment promotes IAB from the helper’s perspective. Study 2 consisted of three experiments. Study 2a examined whether experimentally activating secure attachment would increase positive empathy. Experiment 2b further manipulated participants’ positive empathy (high/low) to test whether higher (vs. lower) positive empathy individuals exhibited more IAB. Experiment 2c tested the mediating effect of positive empathy between secure attachment and IAB by manipulating secure attachment (SAP/non-SAP group). To more comprehensively establish the relationship between positive empathy and IAB, Study 2b experimentally induced positive empathy using video stimuli, thereby providing stronger causal evidence for the effect of positive empathy on IAB. Meanwhile, to verify the robustness of the mediating effect, Experiment 2c manipulated participants’ secure attachment before measuring their positive empathy level and IAB. Thus, the two approaches address distinct questions: Experiment 2b tests the causal effect of positive empathy per se, while Experiment 2c tests its role as a measured mediator. In conclusion, as in the research design of Wang et al. (2023), the present study used both a scale to measure participants’ level of positive empathy and a video to manipulate their positive empathy status to increase the rigor and reliability of the findings.

### 3.1. Study 2a: The Effect of Secure Attachment on Positive Empathy

Experiment 2a used a one-way between-subjects design, with secure attachment (SAP/non-SAP group) as the independent variable and positive empathy as the dependent variable.

#### 3.1.1. Participants

Based on the prior sample size calculations of the G*power 3.1 software, the effect size d = 0.50 with statistical power 1 − *β* = 0.95, two-sided test α = 0.05, and power = 0.80, which gives a sample size of 128. Participants were recruited through advertisements posted on TC Lab, a professional online behavioral research platform widely used in China. The inclusion and exclusion criteria were the same as those in Study 1. A total of 140 participants were recruited through the platform. After excluding 12 incomplete responses, the final sample consisted of 128 participants (57 men, 44.53%). Participants were randomly assigned to the SAP/non-SAP groups (NSAP = 65, Nnon-SAP = 63), with a mean age of 21.79 ± 3.76 years, Internet age of 9.23 ± 3.35 years, and Internet time of 7.32 ± 3.22 h/day.

#### 3.1.2. Measures

SAP: same as study 1. In this study, the Cronbach’s α was 0.93.

Writing validity: same as Study 1.

Positive empathy: The positive empathy scale revised by Yue et al. (2016) was adapted. The scale has seven items (e.g., When I see someone smile, I can’t help but smile back). The scale was scored on 5 points (1 = very inappropriate, 5 = very appropriate), with higher scores indicating higher levels of positive empathy. The Cronbach’s α was 0.78.

#### 3.1.3. Procedures

After arriving at the laboratory classroom, participants were randomly assigned to two different groups (SAP/non-SAP) and first completed a demographic variable survey followed by a recall writing task. After this, participants were instructed to imagine that the recalled person was physically present beside them. Next, participants took the SAP validity measure, completed the writing vividness and ease measures, and finally completed the positive empathy scale. At the end of the experiment, participants received compensation for their participation.

#### 3.1.4. Results

Preliminary analyses. Independent samples *t*-tests revealed a non-significant difference between the SAP group and the non-SAP group for ease of writing (*t*(126) = 0.06, *p* = 0.96) and vividness of writing (*t*(126) = 1.03, *p* = 0.31), which indicated that there was no significant difference in the effectiveness of the writing task between the two groups. The results also showed that the manipulation of the helper’s secure attachment status was valid: there was a significant difference between the SAP group (*M* = 4.39, *SD* = 0.52) and the non-SAP group (*M* = 2.54, *SD* = 0.63), *t*(126) = 17.99, *p* < 0.001, Cohen’s d = 3.19. This very large effect likely reflects the strong contrast generated by the recall-based attachment prime, though caution is warranted because self-generated recall manipulations may amplify perceived differences between conditions. To assess statistical sensitivity, we conducted a sensitivity analysis using GPower 3.1. With a total sample of 128 participants, α = 0.05 (two-tailed), and power = 0.80, the study was sufficiently sensitive to detect an effect size of d = 0.50.

**Figure 2:**
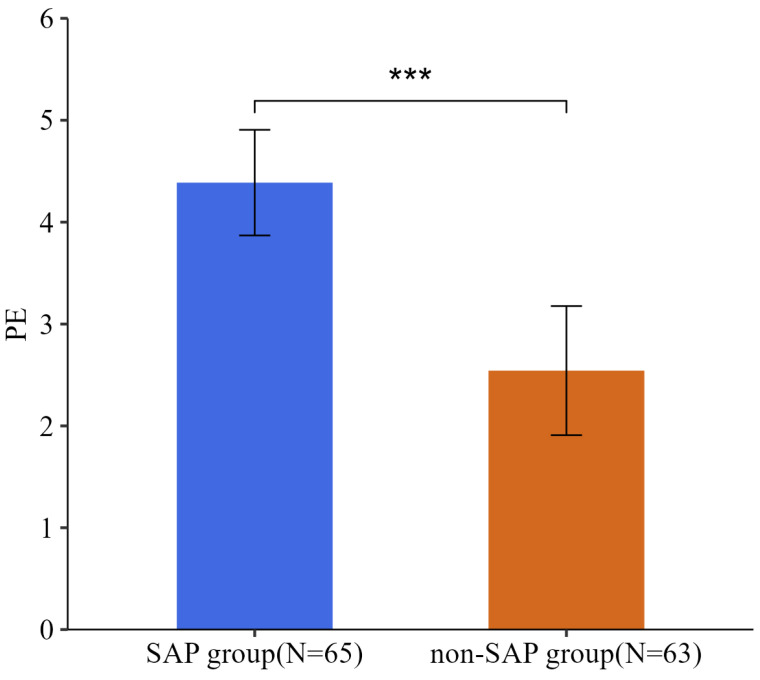
Differences in positive empathy between SAP group and non-SAP group. The blue bar represents the SAP group (N = 65), and the orange bar represents the non-SAP group (control group; N = 63). PE = positive empathy (PE have the same meaning in the next figures). Note. *** *p* < 0.001.

Hypothesis testing. The independent sample *t*-test showed that SAP group (*M* = 3.87, *SD* = 0.52) had higher positive empathy than non-SAP group (*M* = 3.30, *SD* = 0.52), *t*(126) = 6.25, *p* < 0.001, Cohen’s d = 1.10. The results are shown in Figure 2.

### 3.2. Study 2b: The Effect of Positive Empathy on IAB

Experiment 2b utilized a one-way between-subjects design, with positive empathy (high/low) as the independent variable and IAB as the dependent variable.

#### 3.2.1. Pre-Experiments

Positive empathy is both a stable personality quality and an empathic state that can be evoked by stimulus materials. Thus, the primary purpose of the independent pretest was to test the validity of the materials selected to evoke positive empathy. Video-based emotional and empathy induction paradigms have been widely used in psychological research because dynamic audiovisual materials can effectively elicit emotional and empathic responses (e.g., Mayer et al., 2018). In order to effectively activate the participants’ positive empathy, based on previous studies (Li, 2018), this experiment replaced picture priming with video priming, and selected two videos with different positive empathic priming effects (high/low). The high-positive-empathy condition used a video depicting an emotionally uplifting reunion event involving a well-known public figure returning to China after a prolonged legal dispute abroad. The low-positive-empathy condition used a relatively emotionally neutral news commentary video concerning economic regulation and legal governance. To control for the influence of external factors on participants, the duration of the videos, the size of the presented page, and the audio were all consistent in both groups: the video duration was 55 s, the frame width was 720 millimetre, the frame height was 1140 millimetre, and the audio sampling rate was 44.10 kHz.

Through the TC Lab experimental platform, the independent pretest was administered using within-subjects among 85 Chinese college students (33 male students, 38.82%). The two videos were presented to the participants using a randomized order playback. Participants were asked to complete five items after each video they watched, each item was scored using a 7-point scale. Among the five items, Items 1 and 2 are the participants’ evaluations of the selected video (e.g., after watching the video, what was the level of emotion you felt for the characters in the video?); Items 3, 4, and 5 are used to test the validity of positive empathy priming (e.g., after watching the video, do you imagine how the main protagonists would feel in the environment he or she was in?)

The results showed that there was a significant difference in participants’ ratings of the two videos (M high = 6.31, SD = 0.72; M low = 3.95, SD = 1.36; *t*(84) = 15.94, *p* < 0.001; Cohen’s d = 1.73). There were significant differences in participants’ levels of positive empathy after viewing the different videos (M high = 5.02, SD = 0.95; M low = 3.35, SD = 0.64; *t*(84) = 14.50, *p* < 0.001; Cohen’s d = 1.57), which suggests that the selected positive empathy priming materials were appropriate.

#### 3.2.2. Participants

Based on the prior sample size calculations of the G*power 3.1 software, the effect size d = 0.50 with statistical power 1 − *β* = 0.95, two-sided test α = 0.05, and power = 0.80, which gives a sample size of 128. The source of the sample and the screening criteria were the same as in Study 2a. Therefore, this study was released to 160 university students in China through the TC Lab experimental platform, among which 7 data were excluded for the reason that the participants did not answer the questions completely, and finally, valid data were obtained from 153 participants (63 male students, 41.18%). Participants were randomly assigned to the high/low positive empathy groups (Nhigh = 78, Nlow = 75), with a mean age of 21.51 ± 1.64 years, Internet age of 9.07 ± 3.10 years, and Internet time of 7.10 ± 2.78 h/day.

#### 3.2.3. Measures

Positive empathy: same as the pre-experiment of Experiment 2b in Study 2.

IAB: To enhance the robustness of the IAB measurement and enable helpers to experience a more realistic online help-seeking situation, the IAB measurement in this study was carried out by simulation behavior experiment. According to the definition of IAB by Zheng et al. (2011), an Internet help-seeking scenario was set for each of the four dimensions of IAB (Internet guidance, Internet sharing, Internet support, and Internet reminder). These scenarios simulate the help scenes that appear in Zhihu, QQ group, WeChat circle of friends, etc. The four Internet help-seeking scenarios are shown in Supplementary Materials Figure S2. Each scenario was followed by an item (e.g., you saw this message in your WeChat circle of friends, how inclined you are to vote for him/her?), which was used to measure the participant’s IAB. Items were scored on a 7-point scale (1 = never, 7 = most of the time), and the higher the score, the higher the IAB.

#### 3.2.4. Procedures

After entering the experiment, participants were randomly assigned to one of two different positive empathy groups (high/low) and completed the items of the positive empathy priming after watching the video. The instruction was as follows: “The experiment is about to begin, you are required to adjust the volume of your device (appropriate volume is sufficient), watch a video carefully and answer the questions (the video is about 50 s long), and when you are ready, you can enter the experiment by pressing the ‘Start’ button below.”

Participants complete a survey of demographic variables before taking the positive empathy priming, followed by the IAB experiment. At the end of the experiment, participants received compensation for their participation.

#### 3.2.5. Results

Preliminary analyses. An independent samples *t*-test found that the manipulation of positive empathy for the helper was established. There was a significant difference in scores between the high positive empathy group (*M* = 5.36, *SD* = 0.74) and the low positive empathy group (*M* = 3.73, *SD* = 0.67), *t*(152) = 14.36, *p* < 0.001, Cohen’s d = 2.32.

**Figure 3:**
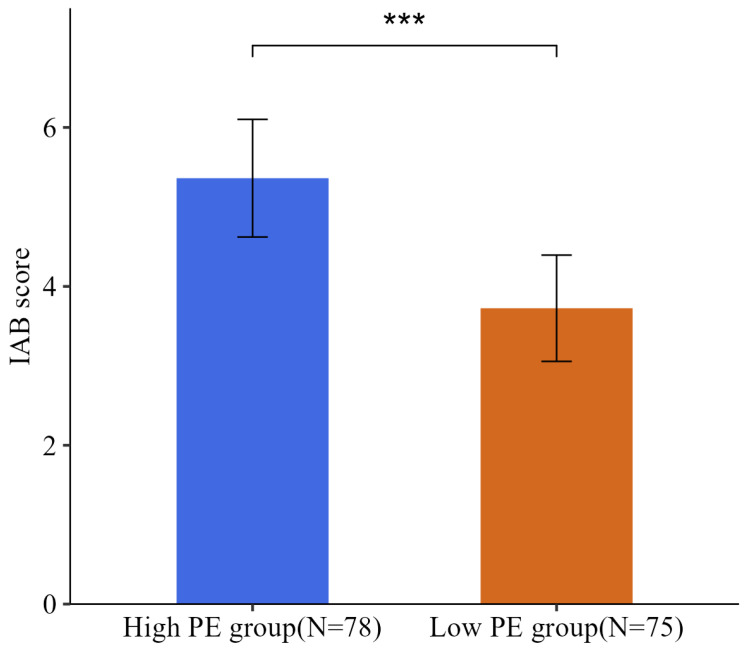
Differences in IAB between high and low PE groups. The blue bar represents the High PE group (N = 78), and the orange bar represents the Low PE group (control group; N = 75). Note. *** *p* < 0.001.

Hypothesis testing. The independent sample *t*-test showed that the IAB score of the high positive empathy group (*M* = 4.86, *SD* = 1.01) was significantly higher than that of the low positive empathy group (*M* = 3.89, *SD* = 0.95). *t*(151) = 6.13, *p* < 0.001, Cohen’ s d = 0.99, indicating that participants in the high-positive-empathy condition exhibited significantly greater IAB than those in the low-positive-empathy condition. The results are shown in Figure 3.

### 3.3. Study 2c: The Effect of Secure Attachment on IAB—The Mediating Role of Positive Empathy

Experiment 2c utilized a one-way between-subjects design, with secure attachment (SAP/non-SAP group) as the independent variable, positive empathy as the mediator, and IAB as the dependent variable.

#### 3.3.1. Participants

Based on the prior sample size calculations of the G*power 3.1 software, the effect size d = 0.50 with statistical power 1 − *β* = 0.95, two-sided test α = 0.05, and power = 0.80, which gives a sample size of 128. The source of the sample and the screening criteria were the same as in Study 1. Thus, 170 paper questionnaires were distributed to university students in China in this study, among which 5 questionnaires were excluded due to missing basic information, resulting in 165 valid questionnaires from participants (72 male students, 43.64%). Participants were randomly assigned to the SAP and non-SAP groups (NSAP = 84, Nnon-SAP = 81), with a mean age of 18.9 ± 0.77 years, Internet age of 8.35 ± 2.79 years, and Internet time of 8.14 ± 3.52 h/day. Previous studies revealed that significant gender differences exist in IAB, and the symptoms of IAB vary among individuals of different ages (Erreygers et al., 2018). Meanwhile, in order to avoid being affected by daily online time, we took gender, age, daily online time, and Internet age as the control variables in this study (Zeng et al., 2020). These variables were also controlled for in Study 3.

#### 3.3.2. Measures

SAP: same as study 1. The Cronbach’s α in this study was 0.96.

Writing validity: same as Study 1.

Positive empathy: same as Experiment 2a in Study 2, the Cronbach’s α was 0.83.

IAB: Same as study 1. In this study, the Cronbach’s α was 0.92.

#### 3.3.3. Procedures

After entering a quiet, comfortable classroom, participants were randomly assigned to two different groups (SAP/non-SAP) and first completed a demographic variable survey followed by a recall writing task. Next, participants were asked to imagine that the person they had just recalled had been next to them. After that, participants took the SAP measure, completed the writing vividness and ease measures, then completed the positive empathy scale followed by the IAB scale, with a short 2 min break inserted between the two scales to reduce potential carryover effects. The order of the scales was fixed for all participants to maintain consistency and control procedural variance. At the end of the experiment, participants received compensation for their participation.

#### 3.3.4. Results

Preliminary analyses. First, common methods bias analysis was carried out. The Harman single-factor test was conducted by confirmatory factor analysis. The results showed that a total of 10 common factors with eigenvalues greater than 1 were proposed, and the first common factor had an explanation rate of 29.62%, less than 40%, indicating that there is no serious problem of common method bias in this study.

Independent samples *t*-tests revealed that IAB in SAP group (*M* = 2.16, *SD* = 0.46) was significantly higher than that in non-SAP group (*M* = 1.71, *SD* = 0.36), *t*(163) = 7.15, *p* < 0.001, Cohen’s d = 1.11. Thus, the results of the present study again validated hypothesis 1. Correlation analysis results (see Table 1) indicated that secure attachment was significantly positively correlated with positive empathy (r = 0.15, *p* < 0.01) and IAB (r = 0.14, *p* < 0.05).

Hypothesis testing. We used Model 4 of the PROCESS macro program (Hayes & Preacher, 2014) to examine the mediating effect of positive empathy on the relationship between secure attachment and IAB when controlling for gender, age, daily online time, and Internet age (see Table 2). Results indicated that secure attachment positively predicted positive empathy (*β* = 0.53, *p* < 0.001, 95% CI = [0.40, 0.66]) and IAB (*β* = 0.52, *p* < 0.001, 95% CI = [0.39, 0.65]). When both secure attachment and positive empathy were entered into the regression equation, secure attachment remained a significant positive predictor of IAB (*β* = 0.34, *p* < 0.001, 95% CI = [0.19, 0.49]). Secure attachment affects IAB through positive empathy with an indirect effect size of 0.18, accounting for 34.62% of the total effect. Next, we used the nonparametric percentile bootstrap method for deviation correction developed by Hayes and Preacher (2014) to test the mediating effect. Under a 95% CI, the CI of the mediating effect of positive empathy was [0.08, 0.30], which did not include 0. These findings partially supported Hypothesis 2, indicating that positive empathy partially mediates the relationship between secure attachment and IAB.

## 4. Study 3: When Does Secure Attachment Affect IAB?—From a Seeker’s Perspective

Study 2 focused on how helper-related factors, particularly secure attachment and positive empathy, contribute to IAB. However, IAB emerges within online interpersonal interactions and is therefore influenced not only by helpers, but also by characteristics of help seekers. Accordingly, Study 3 examined whether seekers’ use of emojis moderates the relationship between helpers’ positive empathy and IAB. This study utilized a 2 (positive empathy: high/low) × 2 (emoji: with/no) between-subjects design, where positive empathy and emoji were the independent variables and IAB was the dependent variable.

### 4.1. Participants

Based on the prior sample size calculations of the G*power 3.1 software, the effect size f = 0.25 with statistical power 1 − *β* = 0.95, two-sided test α = 0.05, and power = 0.80, which gives a sample size of 128. The source of the sample and the screening criteria were the same as in Study 2a. A total of 190 participants were recruited through the TC Lab platform. After excluding nine incomplete responses, the final sample consisted of 181 participants (76 men, 41.99%). Mean age was 21.06 ± 1.79 years, Internet age was 9.41 ± 3.05 years, and Internet time was 7.18 ± 3.05 h/day. One of four groups (Nemoji + high positive empathy group = 46, Nno emoji + high positive empathy group = 44, Nemoji + low positive empathy group = 45, Nno emoji + low positive empathy group = 46).

### 4.2. Measures

Positive empathy: same as Experiment 2b in Study 2.

IAB: same as Experiment 2b.

Emoji: In the emoji condition, emoji symbols were embedded within the online help-seeking messages (see Supplementary Materials Figure S3), whereas the no-emoji condition presented identical messages in plain-text form (see Supplementary Materials Figure S2).

### 4.3. Procedures

Upon entering the experiment, participants were randomly assigned to one of the four experimental conditions. Participants completed a demographic variable survey prior to positive empathy priming, and were then randomly assigned to one of the four groups above, followed by the IAB experiment. At the end of the experiment, participants received compensation for their participation.

### 4.4. Results

#### 4.4.1. Preliminary Analyses

Independent samples *t*-tests found that there was a significant difference in scores between the high positive empathy group (*M* = 4.86, *SD* = 0.92) and the low positive empathy group (*M* = 3.19, *SD* = 0.93), *t*(179) = 12.19, *p* < 0.001, Cohen’s d = 1.81, indicating that the helper’s positive empathy priming was successful.

#### 4.4.2. Hypothesis Testing

**Figure 4:**
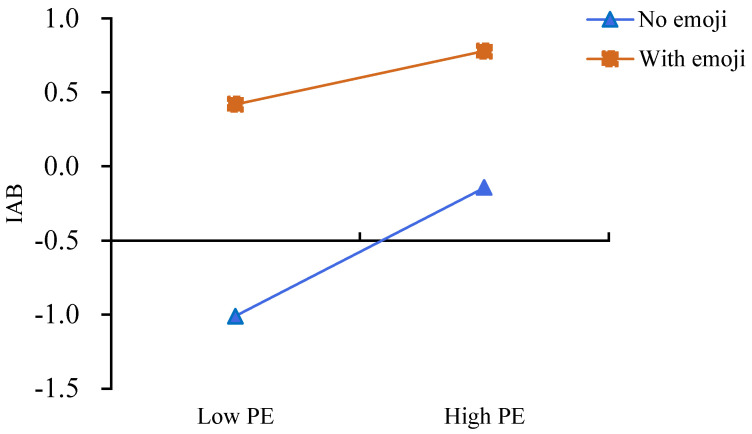
The moderating role of Emoji symbols.

To test Hypothesis 3, the dichotomous moderator variable “emoji” was coded as 0 (no emoji) and 1 (with emoji) for the regression analysis. The independent variable positive empathy and the dependent variable IAB were continuous. We included these variables in PROCESS (Model 1) provided by Hayes and Preacher (2014), and included gender, age, Internet time/day, and Internet age as control variables. The results showed that the product term of positive empathy and emoji significantly predicted IAB (*β* = −0.24, *p* < 0.05, 95% CI = [−0.45, −0.03]), indicating that emoji significantly moderated the relationship between positive empathy and IAB. Simple slope analyses were subsequently conducted to further interpret the interaction effect. The results (see Figure 4) showed that in the presence of emoji, positive empathy significantly predicted IAB (*β* = 0.18, *p* < 0.05, 95% CI = [0.02, 0.34]), and in the no-emoji condition, the positive association between positive empathy and IAB was stronger (*β* = 0.43, *p* < 0.001, 95% CI = [0.28, 0.59]). Thus, Hypothesis 3 was not supported in the predicted direction. Although emoji significantly moderated the relationship between positive empathy and IAB, the observed interaction pattern was opposite to our expectation: emoji weakened rather than strengthened the positive association between positive empathy and IAB.

## 5. Discussion

Previous research has consistently shown that secure attachment facilitates prosocial behavior across a variety of interpersonal contexts (Gross et al., 2017). However, far less is known about whether and how secure attachment promotes IAB, which occurs within digitally mediated social interactions rather than face-to-face contexts. By simulating realistic online help-seeking situations, the present study examined the influence of both helper-related factors and seeker-related cues on IAB, thereby providing a more interaction-based understanding of online prosocial behavior.

### 5.1. The Impact of Secure Attachment on IAB

As hypothesized, this study found that secure attachment could promote the production of IAB, suggesting that the cultivation of secure attachment not only plays an important role in prosocial behavior in the real world, but also affects the occurrence of altruistic behavior in cyberspace. The findings are in line with attachment theory (Bowlby, 1973), which suggests that SAP strengthens an individual’s sense of secure attachment, allowing the helper to believe in his or her abilities to help others and take action. The findings are also consistent with the social-cognitive model of SAP (Baldwin et al., 1996), which proposes that temporary feelings of security may reduce sensitivity to negative social cues while promoting more positive interpersonal responses. For instance, in complex cyberspace, people with a strong sense of secure attachment are less likely to be exposed to unfavorable information, and therefore have more cognitive resources to deal with other network users’ needs for help and respond positively and promptly (Mikulincer & Shaver, 2015). Furthermore, neurological aspects of the brain support the findings of this study, such as the fact that individuals who undergo SAP have lower levels of amygdala activity when confronted with aversive faces (Norman et al., 2015), which is a neurological area of the brain strongly associated with prosocial behavior (Nagamitsu et al., 2010). Another example is that SAP is linked to prefrontal activation, which has been identified as a primary brain area controlling moral behavior (Norman et al., 2015). Taken together, these findings suggest that secure attachment represents an important antecedent of IAB.

### 5.2. The Mediating Role of Positive Empathy

This study found that positive empathy played a significant mediating role between secure attachment and IAB, which is consistent with the hypothesis. On the one hand, secure attachment led to positive empathy. Specifically, SAP not only enhances attachment security, but is also associated with increased positive affect and empathic responding (Cassidy et al., 2018). The heuristic model of the SAP effect (Mikulincer & Shaver, 2007) implies that the activation of secure attachment representations will propagate to the relevant components of sensory perception and emotion management. For example, a study with a group of adolescents found that adolescents who develop secure attachments to their peers develop a positive internal working model of their peers, which contributes to their experience of greater intimacy and emotional support, and therefore higher positive affect (Nickerson & Nagle, 2004). The findings of this study provide empirical support for the hypothesis that secure attachment representations expand to emotional elements. On the other hand, positive empathy also had a promoting effect on IAB. According to the empathy-altruism hypothesis (Batson, 1987), when helpers see someone else struggling and in need of help, they will feel compelled to lend a hand. The stronger their desire to lend a hand and assist the person in need, the more likely it is that helpers will act in an altruistic behavior. Telle and Pfister (2016) also argued that people with high levels of trait positive empathy may have a stronger need to maintain a positive emotional state, and that prosocial behaviors (e.g., IAB) are accompanied by reward experiences that facilitate the maintenance of a positive state. From this point of view, high positive empathizers may engage in IAB in order to satisfy the need to maintain a positive emotional state. Empirical studies also show that empathy significantly and positively predicts IAB (Zheng & Zhao, 2015). Taken together, secure attachment stimulates greater positive emotional experiences, producing positive empathy, which in turn exhibits IAB.

It is noteworthy that the mediation effect was partial, with positive empathy accounting for 34.62% of the total effect of secure attachment on IAB. The partial mediation observed in the present study suggests that additional psychological mechanisms may also account for the association between secure attachment and IAB. Beyond positive empathy, individuals with a stronger sense of secure attachment may be more likely to trust others in online environments (Shaver et al., 2019), perceiving less risk and more reliability in anonymous interactions, which could directly foster prosocial responses. They may also possess higher self-efficacy in their ability to provide effective help online (F. Yang & Oshio, 2025), believing their actions can make a difference. Future research that simultaneously tests these parallel mediators alongside positive empathy would provide a more comprehensive understanding of the multifaceted motivational pathways from secure attachment to online altruism.

### 5.3. The Moderating Role of Emoji

The results showed that the interaction between positive empathy and emoji significantly predicted IAB, indicating that emoji moderated the relationship between positive empathy and IAB. This finding is consistent with symbolic interactionism (Blumer, 1969), which proposes that individuals communicate and respond to others through socially meaningful symbols. In online help-seeking contexts, seekers may use emoji to convey emotions and intentions, whereas helpers interpret these cues and adjust their responses accordingly.

Contrary to our hypothesis, however, positive empathy predicted IAB more strongly in the no-emoji condition than in the emoji condition. This pattern is broadly consistent with the antagonistic interactions model of moderation (Cohen et al., 2003), in which one protective factor weakens the effect of another protective factor on an adaptive outcome. One possible explanation is that emoji function as salient affective cues that increase the emotional clarity of help-seeking messages, particularly for individuals lower in positive empathy. As a result, these individuals may become more willing to help when emoji are present, thereby reducing the relative advantage typically shown by highly empathic individuals. One possible explanation is that, because emotional information is already externally conveyed through emoji, helpers may rely less heavily on their own empathic tendencies when deciding whether to help.

Variation in emoji type may also have contributed to the weaker-than-expected moderating effect. Although both facial and non-facial emoji were included to enhance ecological validity (Riordan, 2017), previous studies suggest that facial emoji may communicate emotions more effectively than non-facial symbols (Jaeger et al., 2019). Future research may benefit from distinguishing different emoji categories and examining their specific interpersonal functions.

Importantly, positive empathy remained a significant predictor of IAB even in the emoji condition, and overall IAB was higher in the emoji group than in the no-emoji group. This suggests that emoji still play an important facilitating role in online interaction. Emoji may enhance emotional expression (Gesselman et al., 2019), increase communication enjoyment, and strengthen interpersonal closeness (Holtgraves & Robinson, 2020), all of which may increase the likelihood of receiving help online.

### 5.4. Implications

This study offers several theoretical contributions. Most importantly, the findings suggest that IAB may not simply represent an online extension of offline prosociality, but rather a form of prosocial behavior shaped by digitally mediated social cues. By integrating helper-related factors (secure attachment and positive empathy) with seeker-related cues (emoji use), the present research highlights how online helping behaviors emerge through the dynamic interplay between individual dispositions and contextualized digital communication signals. In doing so, the study extends attachment theory (Bowlby, 1973) into the domain of online interpersonal interaction and provides a more interaction-based framework for understanding IAB. Second, positive empathy may be a mediating variable of the influence of secure attachment on IAB, which provides a new idea for exploring the mechanisms by which IAB occurs. Third, this study validates the social information-processing models (Crick & Dodge, 1994) by finding the moderating effect of emoji symbols on the relationship between positive empathy and IAB.

Practical implications drawn from our study are also particularly important. This study confirms that initiating an individual’s secure attachment based on the core components of secure attachment and attempting to create an environment that makes a person feel warmer and safer is of great practical significance in increasing an individual’s IAB and promoting harmonious interpersonal relationships in cyberspace. Second, cultivating personal positive empathy in education and social practice is not only conducive to enhancing people’s happiness in real life, but also the key to promoting people’s positive behavior in cyberspace. In addition, encouraging users to use emoji in online communication, especially in help-seeking situations, can inspire others to behave positively in cyberspace; in other words, making use of emoji opens up more possibilities for IAB to occur.

Finally, while the present study focused primarily on attachment-related psychological mechanisms, individual differences such as gender may also shape sensitivity to social cues, emotional responsiveness, and online helping behaviors. Future research could systematically examine whether gender moderates the relationships among secure attachment, positive empathy, emoji processing, and Internet altruistic behavior, thereby providing a more nuanced understanding of online prosocial interaction.

### 5.5. Limitations and Future Research

Although the present research employed multiple studies and complementary methods to strengthen internal validity, several limitations should nevertheless be acknowledged when interpreting the findings.

First, the generalizability of the results may be constrained by the exclusive reliance on Chinese university students, who represent a relatively specific developmental and sociocultural group embedded in highly digitalized interaction contexts. Consequently, the observed mechanisms may not generalize directly to other age groups or cultural settings. Future research should examine whether the proposed relationships replicate across broader populations and sociocultural environments.

Second, positive empathy only partially mediated the relationship between secure attachment and IAB, indicating that additional psychological mechanisms may also be involved. Variables such as interpersonal trust, prosocial self-efficacy, or moral identity may help explain the remaining direct effect and deserve further investigation.

Third, IAB was assessed using two different methods across studies: a self-report scale and scenario-based behavioral simulations. Although each method was selected to match the goals of specific studies and both demonstrated acceptable utility, measurement heterogeneity may have introduced additional variance. Future research could administer multiple indicators within the same sample to establish stronger convergent validity.

Fourth, some experimental stimuli warrant caution. In Study 2b, the video prime depicting Meng Wanzhou’s return to China may have elicited not only positive empathy, but also broader collective emotions, such as patriotic identification, collective pride, or social identification associated with a culturally symbolic event. Thus, the construct validity of this manipulation should be interpreted cautiously. Future studies should employ more politically neutral and standardized emotional stimuli. Similarly, one scenario in the scenario-based IAB measure included a socially and ideologically resonant slogan, which may have activated motivations beyond altruism (e.g., social conformity or identity-related responses). Although the scenario was designed to represent the “Internet support” dimension of the established IAB framework (Zheng et al., 2011), future studies may further refine scenario wording to minimize potential construct-irrelevant influences. In Study 3, different emoji categories and placement positions may also influence interpersonal perceptions and helping responses. Future work may systematically examine these boundary conditions.

Fifth, several methodological considerations remain. Common method bias in Study 2c cannot be fully ruled out because key variables were self-reported within a single session. In addition, mixed-mode data collection and the absence of formal pre-registration may have reduced methodological transparency. Future studies could adopt temporally separated measures, unified data-collection procedures, behavioral outcomes, and pre-registered confirmatory designs to strengthen confidence in the robustness and replicability of the findings.

## 6. Conclusions

The present study extends the literature on IAB by examining its underlying mechanisms from the dual perspectives of helpers and seekers within realistic online interaction contexts. Across three studies, the findings consistently demonstrated that secure attachment positively promotes IAB, both directly and indirectly through positive empathy. Specifically, positive empathy partially mediated the relationship between secure attachment and IAB, suggesting that individuals with stronger attachment security may be more likely to engage in online helping behaviors because they experience greater positive emotional resonance with others.

In addition, the present study further revealed that seekers’ use of emoji constitutes an important contextual factor shaping online altruistic interactions. Although the moderating effect was opposite to our original prediction, emoji significantly influenced the relationship between positive empathy and IAB, highlighting the complex role of digital emotional cues in cyberspace. Overall, the findings contribute to a more comprehensive understanding of how attachment-related psychological processes and online social cues jointly shape altruistic behavior in digital environments.

## Figures and Tables

**Table 1:** Descriptive statistics and intercorrelations between variables.

Variables	*M*	*SD*	1	2	3	4	5	6	7
1. Gender	0.25	0.44	1						
2. Age	18.93	0.77	0.10	1					
3. Online time/day	8.14	3.52	0.12	−0.18 *	1				
4. Internet age	8.35	2.79	0.03	−0.03	0.10	1			
5. Secure attachment	3.39	1.15	−0.17 *	0.03	−0.05	0.04	1		
6. Positive empathy	3.31	0.72	−0.19 *	−0.09	0.04	−0.05	0.54 ***	1	
7. IAB	1.94	0.47	−0.12	−0.10	−0.04	−0.05	0.52 ***	0.53 ***	1

Note. * *p* < 0.05. *** *p* < 0.001. Virtual encoding for gender, Male = 1, Female = 0.

**Table 2:** Mediating effect model.

Predictor Variables	Model 1 (IAB)			Model 2 (Positive Empathy)			Model 3 (IAB)			VIF (Model 3)
	*β*	*SE*	*t*	*β*	*SE*	*t*	*β*	*SE*	*t*	
Gender	−0.04	0.16	−0.28	−0.23	0.15	−1.48	0.03	0.15	0.22	1.07
Age	−0.15	0.09	−1.68	−1.11	0.09	−1.27	−0.11	0.08	−1.32	1.06
Online time/day	−0.01	0.02	−0.35	0.02	0.02	1.16	−0.01	0.02	−0.77	1.02
Internet age	−0.03	0.02	−1.11	−0.03	0.02	−1.25	−0.02	0.02	−0.73	1.08
Secure attachment	0.52	0.07	7.66 ***	0.53	0.07	8.05 ***	0.34	0.08	4.44 ***	1.45
Positive empathy							0.34	0.08	4.43 ***	1.48
*R* ^2^	0.29			0.32			0.37			
*F*	12.92 ***			15.17 ***			15.30 ***			

*** *p* < 0.001.

## Data Availability

The data presented in this study are openly available in [https://osf.io/ynh5k/?view_only=6a35f705a26046a7a5691dcdfe05aea1] (accessed on 20 May 2026).

## Supplementary Materials

The following supporting information can be downloaded at: https://www.mdpi.com/article/10.3390/bs16060877/s1, Figure S1: Hypothetical models; Figure S2: Internet help-seeking scenarios; Figure S3: Internet help-seeking scenarios (with emoji symbols).
