# Supplementary material for: The Impact of Secure Attachment on Internet Altruistic Behavior: From the Helper and Seeker’s Perspective
Source: Behav Sci (Basel). 2026 Jun 1;16(6):877. doi: 10.3390/bs16060877 (PMC13295773; doi:10.3390/bs16060877)
Supplement: Supplementary file 1 [file behavsci-16-00877-s001.zip › behavsci-4246351-supplementary.pdf]

Supplementary Material

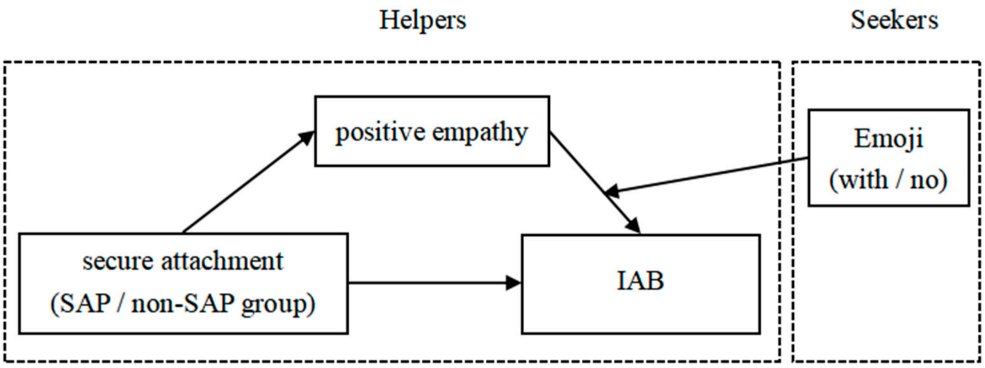

Supplementary Material Figure S1. Hypothetical models. IAB = Internet altruistic behavior, and SAP = secure attachment priming (IAB and SAP have the same meaning in the next tables and figures).

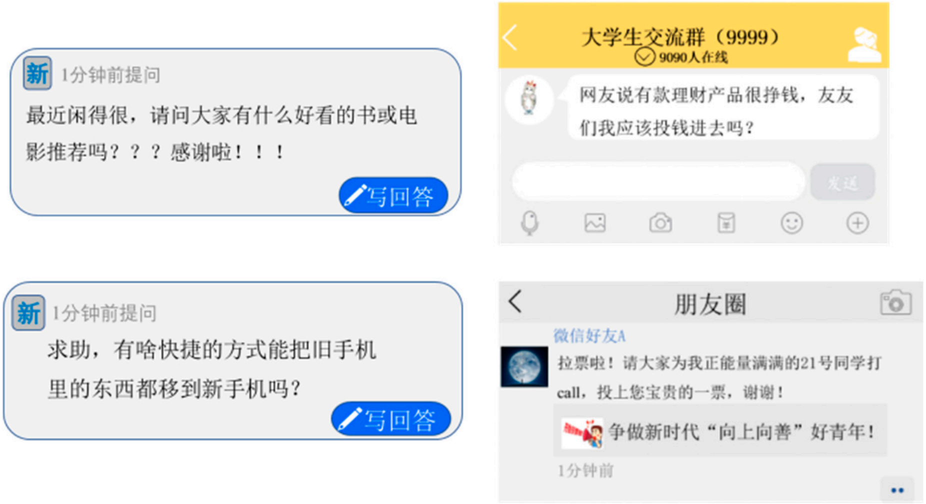

Supplementary Material Figure S2. Internet help-seeking scenarios.

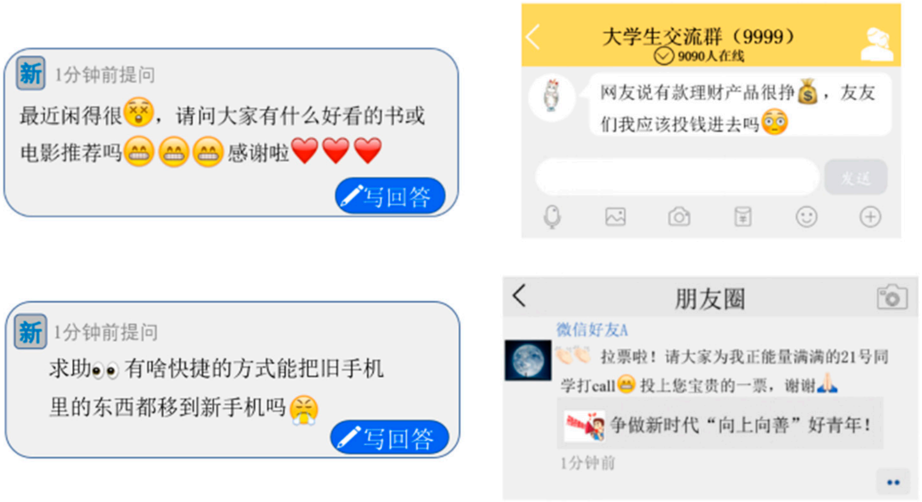

Supplementary Material Figure S3. Internet help-seeking scenarios (with emoji symbols).
